# Bioprinting of 3D Convoluted Renal Proximal Tubules on Perfusable Chips

**DOI:** 10.1038/srep34845

**Published:** 2016-10-11

**Authors:** Kimberly A. Homan, David B. Kolesky, Mark A. Skylar-Scott, Jessica Herrmann, Humphrey Obuobi, Annie Moisan, Jennifer A. Lewis

**Affiliations:** 1 School of Engineering and Applied Sciences, Wyss Institute for Biologically Inspired Engineering, Harvard University, Cambridge, Massachusetts, United States of America; 2 Pharmaceutical Sciences, Roche Pharma Research and Early Development, Roche Innovation Center, Basel, Switzerland

## Abstract

Three-dimensional models of kidney tissue that recapitulate human responses are needed for drug screening, disease modeling, and, ultimately, kidney organ engineering. Here, we report a bioprinting method for creating 3D human renal proximal tubules *in vitro* that are fully embedded within an extracellular matrix and housed in perfusable tissue chips, allowing them to be maintained for greater than two months. Their convoluted tubular architecture is circumscribed by proximal tubule epithelial cells and actively perfused through the open lumen. These engineered 3D proximal tubules on chip exhibit significantly enhanced epithelial morphology and functional properties relative to the same cells grown on 2D controls with or without perfusion. Upon introducing the nephrotoxin, Cyclosporine A, the epithelial barrier is disrupted in a dose-dependent manner. Our bioprinting method provides a new route for programmably fabricating advanced human kidney tissue models on demand.

Engineering human tissues, and ultimately organs, that recapitulate native function for use in drug screening, disease modeling, and regenerative medicine is a grand challenge. Incidence rates of chronic and acute kidney injury are spiking due to increased use of prescription drugs^1^,^2^,^3^. Although roughly 25% of acute renal failure observed in the clinic is drug induced^2^, predicting nephrotoxicity in preclinical *in vitro* or animal studies remains difficult. In fact, renal toxicity accounts for only 2% of failures in preclinical drug testing, yet it is responsible for nearly 20% of failures in Phase III clinical trials^3^,^4^,^5^. Hence, there is a critical need for improved kidney tissue models that can both predict human drug toxicity in longitudinal preclinical testing and serve as a modular building block for engineering human nephrons and, ultimately, kidneys.

While renal injury can occur in many locations, including the renal vascular network, glomerulus, tubulointerstitium, and collecting ducts, the convoluted proximal tubule (PT) is the site most frequently damaged (Fig. 1a) 1. The PT is responsible for 65–80% of nutrient absorption and transport from the renal filtrate to the blood, and thus, circulating drugs and their metabolites often accumulate in the PT at high concentrations in both intra- and intercellular spaces. Unfortunately, compared to their *in vivo* counterparts, proximal tubule cells grown in traditional 2D cell culture often lack, or rapidly lose, key phenotypic and functional aspects such as cell polarity, apical brush border, and significant receptor-mediated transport, hindering accurate longitudinal predictions of *in vivo* nephrotoxicity^6^. *In vitro* models that recapitulate the *in vivo* phenotype and function of proximal tubule cells could lead to more predictive nephrotoxicity models.

Towards this objective, several kidney PT models have been developed^7^. Proximal tubule cells have been cultured on biomimetic basement membrane coatings or on hollow fibers^8^,^9^,^10^,^11^, improving their proliferation and ability to self-organize and maintain a differentiated state^12^,^13^,^14^. Researchers have also attempted to recreate the complex 3D microenvironments of the kidney. For example, differentiated proximal tubule cells have been shown to assemble into 3D structures within thin gels^15^,^16^, and, more recently, induced pluripotent stem cell-derived kidney organoids have been created that contain various nephronal features^17^,^18^,^19^,^20^,^21^. While the emerging tissue complexity is compelling, kidney organoids are limited to roughly one millimeter in size and lack addressable inlet and outlets. Hence, proximal tubules within these organoids cannot be directly probed, nor can their perfusate be easily collected and analyzed. To date, perfusion has only been achieved within kidney-on-a-chip devices, which consist of a single layer of proximal tubule cells seeded on a porous membrane^22^. Despite their planar arrangement, the proximal tubule cells in these devices are subjected to a controlled shear stress environment^23^ that significantly enhances their differentiated state as well as their response to nephrotoxic drugs. However, each of these existing models lack one or more characteristic features, i.e., 3D convolution, open luminal architecture, perfusion at physiological shear stresses, and longevity^7^,^24^, required to achieve a truly biomimetic PT model.

One emerging approach suitable for producing complex, luminal tissue architectures is 3D bioprinting, which we originally developed for vascularized human tissues^25^,^26^. Here, we report a method that combines bioprinting, 3D cell culture, and organ-on-a-chip concepts to create a 3D convoluted renal proximal tubule (PT) composed of a perfusable open lumen that possesses a programmable architecture, which can support extratubular cellular heterogeneity. These 3D convoluted PTs consist of an open lumen architecture circumscribed by proximal tubule epithelial cells (PTECs), embedded in an extracellular matrix, and housed within a perfusable tissue chip, where they are subjected to physiological shear stresses. PTECs form a confluent epithelial monolayer that exhibits primary cilia and expresses Na^+^/K^+^ ATPase, Aquaporin 1 (AQP1), and K cadherin. Furthermore, cytokines produced by PTECs can be analyzed by collecting tubule perfusate. The unique combination of their 3D geometry and controlled perfusion gives rise to a more differentiated, polarized PTEC phenotype that develops an enhanced brush border, basement membrane protein deposition, basolateral interdigitations, enhanced cell height, megalin expression, and albumin uptake relative to both perfused and non-perfused 2D controls. The effects of the nephrotoxin, cyclosporine A, are analyzed by directly imaging as well as quantifying the diffusional permeability of the epithelium. To our knowledge, this is the first demonstration of bioprinted 3D convoluted proximal tubules with an addressable open lumen that can be maintained longitudinally.

## Results

### Printing, Seeding, and Longitudinal Culture of 3D Proximal Tubules on Chip

**Figure 1:**
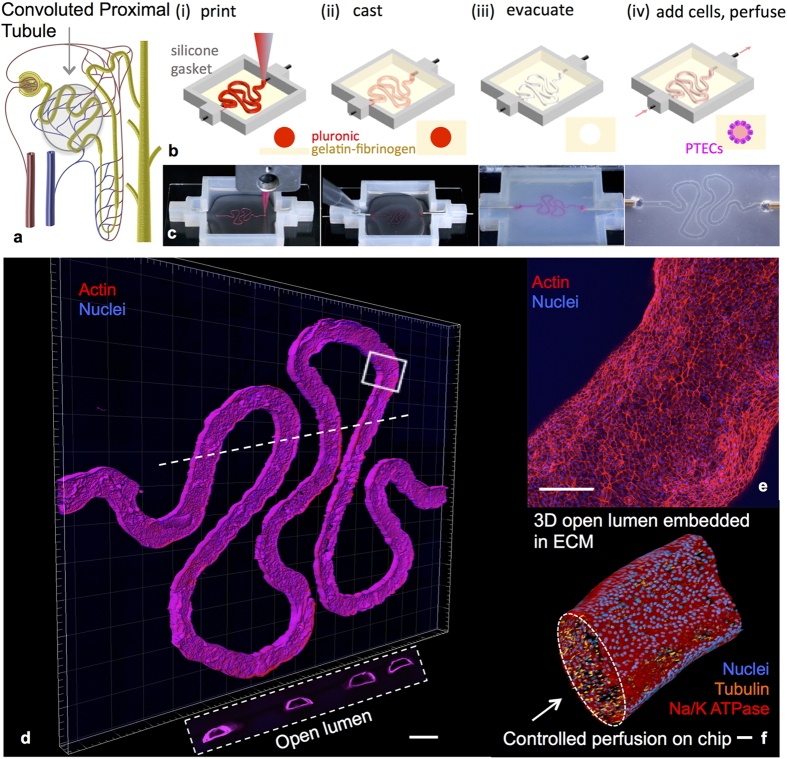
3D convoluted renal proximal tubule on chip. **(a)** Schematic of a nephron highlighting the convoluted proximal tubule, (**b,c**) corresponding schematics and images of different steps in the fabrication of 3D convoluted, perfusable proximal tubules, in which a fugitive ink is first printed on a gelatin-fibrinogen extracellular matrix (ECM) (i), additional ECM is cast around the printed feature (ii), the fugitive ink is evacuated to create an open tubule (iii), and PTEC cells are seeded within the tubule and perfused for long time periods (iv); (**d**) a 3D rendering of the printed convoluted proximal tubule acquired by confocal microscopy, where actin is stained in red and nuclei are blue; the white dotted line denotes the location of the cross-sectional view shown below in which PTEC cells circumscribe the open lumens in 3D, scale bar = 500 μm, (**e**) higher magnification view of the region in (**d**) denoted by the white rectangle, scale bar = 200 μm, (**f**) 3D rendering of the convoluted renal proximal tubule where an open lumen circumscribed with an epithelial lining is directionally perfused on chip and Na/K ATPase is stained in red, acetylated tubulin is orange highlighting the primary cilia, and nuclei are blue, scale bar = 50 μm.

Our bioprinting method is used to construct a 3D convoluted proximal tubule segment of a nephron, as depicted in Fig. 1a 26. First, as shown in Fig. 1b,c (and Movie S1), a silicone gasket is printed on a glass slide that demarcates the outer border of the 3D tissue chip. A layer of engineered extracellular matrix (ECM), which is composed of a gelatin-fibrin hydrogel^25^, is then evenly deposited within the gasket. Next, a fugitive ink, shown in pink, is printed onto the ECM layer. The term “fugitive ink” refers to a printed material that will ultimately be liquefied and removed from the final 3D PT construct. After printing, the fugitive ink is connected to hollow metal pins interfaced through the gasket walls and additional ECM is cast over the printed structure. The 3D tissue model is then housed within a perfusable chip, where it is cooled to 4 °C to liquefy and subsequently remove the fugitive ink yielding an open convoluted tubular channel embedded within the ECM. Finally, cell media is perfused through the 3D convoluted tubular architecture on chip via an external peristaltic pump. Notably, our method can create 3D proximal tubule models in myriad configurations with precisely controlled size, curvature, and location. For instance, if multiple tubules are required to increase statistical relevance of an assay or provide basal-side access channels, they can be printed alongside one another (Fig. S1) and either perfused independently or collectively through a single inlet.

The composition and rheological properties of the ECM and fugitive ink are specifically tailored for our biofabrication method. The ECM consists of fibrinogen, gelatin, and two enzymes (thrombin and transglutaminase)^25^. The dual enzyme scheme enables rapid solidification of the ECM around printed features, through thrombin action on fibrinogen to make fibrin. The second enzyme, transglutaminase, provides a slower crosslinking of gelatin with fibrin, enabling a seamless integration of the upper and lower ECM layers during assembly (Fig. S2a). Furthermore, the elastic modulus of the ECM (~3.5 kPa) mimics that of the cortex of a healthy kidney (~4 kPa)^27^; both matrix stiffness and composition are important for the retention of tissue-specific cell functionality^12^,^28^. The fugitive ink is composed of a triblock copolymer of polyethylene-polypropylene-polyethylene (Pluronic^®^ F127), which forms a viscoelastic gel above a critical micelle concentration in water at room temperature. This ink exhibits a gel-to-fluid transition as the perfusable tissue chip is cooled to 4 °C, enabling its removal from the ECM under those conditions^26^,^29^. The fugitive ink also contains a high concentration of thrombin (100 U/mL). Upon surrounding this ink with ECM during the casting process, soluble fibrinogen is rapidly transformed to insoluble fibrin, templating fibrin around the lumen and facilitating the desired, long-term perfusion of cell media.

Prior to introducing cells, we perfuse the 3D tissue chip with cell media overnight at 37 °C to remove any residual fugitive ink or enzymes and equilibrate the matrix at 37 °C and 5% CO_2_ in the incubator. We then introduce PTEC-TERT1 cells that consist of human proximal tubular cells immortalized through stable expression of the catalytic subunit of human telomerase reverse transcriptase (*TERT*)^30^. PTEC-TERT1 were developed as a cell model that maintains morphological and functional properties of primary PTEC cells with an additional replicative advantage over primary cells that have a finite lifespan *in vitro* due to telomere shortening^16^,^30^. Genomic stability of PTEC-TERT1 up to 90 population doublings has been demonstrated^30^. We further profiled PTEC-TERT1 by carrying out gene expression analysis on 33 key PTEC genes and comparing them with primary PTEC and the renal cancer cell line A498 (Fig. S2b). The mRNA levels demonstrate that PTEC-TERT1 cells are transcriptionally close to primary renal PTEC cells. Given the need for scalable, stable cellular systems in drug discovery and safety platforms, we optimized our 3D PT model with PTEC-TERT1 (hereby referred to as PTECs).

To circumscribe the convoluted tubules with a confluent PTEC monolayer, the cells are first trypsinized from a tissue culture plastic dish, concentrated, and perfused into the open lumen of the printed structure. The cells incubate in the tubule overnight with no flow to facilitate adherence to the ECM and are then flushed lightly at Day 1 to remove any non-adherent cells. A time sequence of their maturation process in the tubule is provided (Fig. S3). Notably, PTECs grow to confluency within the tubule, circumscribing the open lumen in 3D over a period of approximately 3 weeks. Furthermore, since PTECs actively participate in pro-inflammatory cytokine production *in vivo* and *in vitro*^1^,^31^, we measured the accumulation of IL6, IL-8 and MCP1 in the tubule perfusate over time. The cytokine profile shows distinct concentrations in the growth and maturation phase, suggesting the tubule stabilizes after confluency (Fig. S4). Moreover, the decrease of Il-6 concentration after serum removal is consistent with the previously reported inductive effect of albumin on IL-6 production in primary human PTECs^32^.

For increasing levels of complexity, support cells, such as fibroblasts or immune cells, can be suspended in the ECM surrounding the printed tubules^25^,^26^. As shown in Fig. S5, fibroblasts can survive adjacent to the tubule in the extratubular space of the ECM. While tubule diameters ranging from 150 μm to 700 μm can be printed, we carried out assays and quantitative measurements on PTs with diameters ranging from 400 μm to 550 μm under a flow rate of ~1 μL/min. Images of a mature PT at low and higher magnifications (Fig. 1d–f) reveal that PTECs circumscribe the lumen and adopt a cuboidal morphology, as expected for their *in vivo* phenotype. These engineered 3D convoluted PTs are maintained longitudinally by perfusing media in a closed-loop system. Media is replaced every two days and the tubules remain viable for extended periods; the longest period tested exceeds two months (65 days).

### 3D Proximal Tubules form a Polarized Epithelium

**Figure 2:**
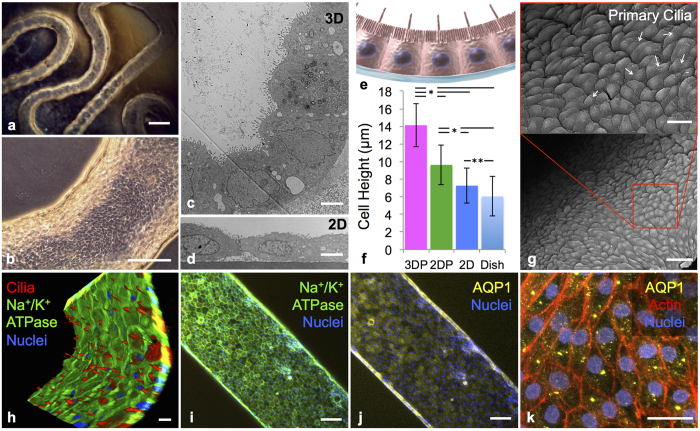
3D proximal tubule morphology and molecular markers. **(a)** A phase contrast image of a mature 3D PT construct taken at 6 weeks, scale bar = 500 μm, (**b**) phase contrast image of the 3D PT construct at 6 weeks, scale bar = 250 μm, (**c**) TEM image of the PTECs within the tubule at 5 weeks, scale bar = 5 μm, (**d**) TEM image of the PTECs grown on a 2D dish coated with ECM with no perfusion, scale bar = 5 μm, (**e**) schematic view of the columnar epithelium seen in native tissue, in which PTECs pack together closely and exhibit a dense brush border on the apical side, tight junctions, and a solid basement membrane, (**f**) PTEC cell height as measured from TEM images of the 3D PT constructs (3DP) as well as three 2D controls (2DP = PTECs on ECM in 2D with perfusion, 2D = PTECs on ECM in 2D not perfused, Dish = bare tissue culture dish not perfused), *p < 0.001, **p < 0.02, (**g**) SEM images at low (scale bar = 50 μm) and higher (scale bar = 20 μm) magnifications showing a confluent layer of PTECs within the 3D PT, white arrows highlight the presence of primary cilia at a density of one per cell, (**h**) 3D rendering of a partial tubule showing the apical side, which highlights the primary cilia (red), scale bar = 20 μm, (**i**) image of the PT highlighting the presence of Na/K ATPase in green, scale bar = 100 μm, (**j**) image of the 3D PT highlighting the presence of AQP1 in yellow, scale bar = 100 μm, (**k**) high magnification view of the image in (**j**) highlighting actin in red and showing AQP1 in yellow, scale bar = 20 μm.

**Figure 3:**
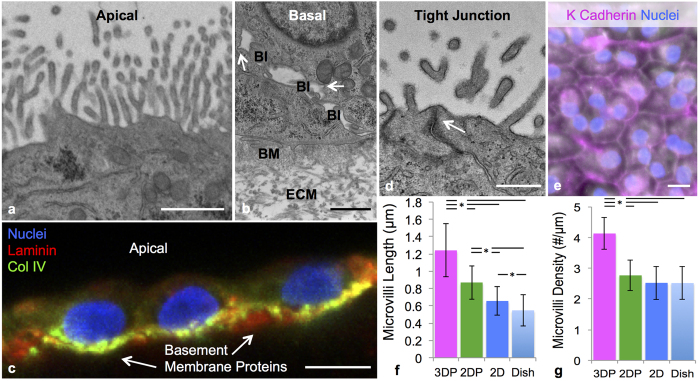
3D proximal tubules form a tissue-like polarized epithelium. **(a)** TEM image of the brush border on the apical side of PTECs at 6 weeks, scale bar = 1 μm, (**b**) TEM image of the basal side of PTECs at 6 weeks highlighting the presence of the engineered extracellular matrix (ECM), basement membrane proteins secreted by the cells (BM), basolateral interdigitations (BI), and circular invaginations in the membrane marked with white arrows, scale bar = 1 μm, (**c**) PTECs at 6 weeks showing the basement membrane proteins the cells secreted, namely laminin (predominant protein in red) and collagen IV (green), scale bar = 10 μm, (**d**) tight junction (white arrow) between PTECs in the bioprinted tubule, scale bar = 500 nm, (**e**) the cell junction protein K Cadherin (magenta) stained in the PT, scale bar = 10 μm, (**f**) microvilli length and (**g**) microvilli density quantified through TEM images of the 3D PT constructs (3DP) as well as three 2D controls (2DP = PTECs on ECM in 2D with perfusion, 2D = PTECs on ECM in 2D without perfusion, Dish = bare tissue culture dish without perfusion), p < 0.001.

After PTECs are seeded and grown to maturity in the tubule, a combination of light microscopy, scanning electron microscopy (SEM) and transmission electron microscopy (TEM) are used to characterize the printed and perfused 3D PT (Figs 2 and 3). Specifically, low (Fig. 2a) and high (Fig. 2b) magnification views in phase microscopy reveal that PTECs grow throughout the tubule packing together in a columnar fashion. TEM images of the tubule cross-section further show that PTECs assemble into a tightly packed, columnar renal tubular epithelium (Fig. 2c,d). As shown schematically in Fig. 2e, native epithelium forms a basement membrane on the basal side and a brush border of microvilli on the apical side facing the open lumen with cells in a columnar morphology. From the TEM images, we quantified the increase in cell height, owing to the columnar cell morphology within the 3D proximal tubule (Fig. 2c) compared to the same cells grown for the same duration in 2D on ECM without perfusion (Fig. 2d). Importantly, the PTECs in our printed and perfused 3D PT constructs exhibit a two-fold increase in cell height relative to the planar controls without perfusion and a 40% increase relative to perfused 2D controls on our ECM (Fig. 2f). Moreover, the cell height of 14.1 ± 2.4 μm observed in our 3D PT constructs approaches that found in healthy human proximal tubules (20.3 ± 4.1 μm).

SEM images of the apical side of the 3D PT (Fig. 2g) reveal the formation of a confluent cell layer and the presence of primary cilia (one per cell, akin to that observed *in vivo*). The primary cilium is a sensory organelle that extends into the open lumen and responds to shear stress; it is important for the maintenance of the epithelial cell phenotype and is often lost once cells are isolated and cultured in 2D in the absence of shear stress^22^. Primary cilia are also observed in our PT using immunofluorescence, by staining for acetylated tubulin (shown in red in the 3D rendering in Figs 2h and S6). Furthermore, we confirmed the expression of the epithelial marker Na^+^/K^+^ATPase (Fig. 2h,i), and its appropriate sub-cellular localization to the basolateral plasma membrane (Fig. S6a), which is again akin to the *in vivo* PTEC phenotype. The proximal tubule-specific (versus distal tubule) water channel Aquaporin 1 (AQP1) is also predominant throughout our tubule (Fig. 2j) and the AQP1 staining at higher magnification has a speckled pattern on the membrane surface (Fig. 2k) as others have shown^33^. We also observe proper apical expression of lotus tetragonolobus lectin (LTL) (Fig. S6c) and basal expression of organic cation transporter (OCT2) (Fig. S6d).

Cell polarity is a fundamental feature needed for vectorial transport. We explored PTEC polarity by first characterizing the apical side of our 3D PT using TEM (Fig. 3a). At the apical surface, microvilli are present and form a brush border that is more pronounced than in 2D (compare Figs 2c and 3a with Fig. 2d). At the basal (Fig. 3b) surface, basolateral interdigitations (BI) are prominent. These BI extend the surface area of the lateral and basal borders *in vivo*. By contrast, PTEC cells in the 2D controls (Fig. 2d) lack BI. The presence of circular invaginations in the lateral membrane, denoted by white arrows in Fig. 3b, suggest that mechanisms of active transport are present at the lateral surface. Furthermore, there is a distinct difference between the ECM morphology and basement membrane (BM) proteins deposited by the PTECs. Further exploration of the BM protein composition reveals that in mature 3D PT constructs, PTECs deposit laminin and collagen IV (Fig. 3c). Tight junctions between neighboring cells are observed (Fig. 3d) along with the presence of cell-cell junction proteins, such as K cadherin in Fig. 3e, that link cells in a characteristic cobblestone pattern. Lastly, properties of the brush border are quantified by image analysis. We find that the average microvilli length in the 3D printed and perfused PTs is ~200% longer than the 2D non-perfused and ~40% higher than the 2D perfused controls (Fig. 3f). Concurrently, microvilli density is also significantly higher for the printed and perfused 3D PT constructs compared to all 2D control conditions (all 2D controls are statistically similar) (Fig. 3g). Once again, the microvilli length (1.24 ± 0.3 μm) and density (4.13 ± 0.5 μm) observed in our 3D PT constructs is closer to that of healthy human proximal tubules, which are 2.89 ± 0.6 μm and 7.81 ± 1.0/μm, respectively.

PTECs should form near leak tight barriers against the traffic of certain proteins, like low molecular weight inulin, when healthy and confluent. To assess their barrier function^34^, we perfused FITC-labeled inulin (4.5 kDa) through the open lumen of mature PTs and the measured dye intensity using a wide-field fluorescence scope as a function of time. From these data, we determined the diffusional permeability and compared this value to a control measurement carried out in a 3D tubule without epithelial lining (Fig. S7). We observed a dramatic reduction in the diffusional permeability coefficient (greater than an order of magnitude) between these two samples, indicating that the epithelial barrier in the printed and perfused 3D PT construct is tight and functional.

### Albumin Uptake

**Figure 4:**
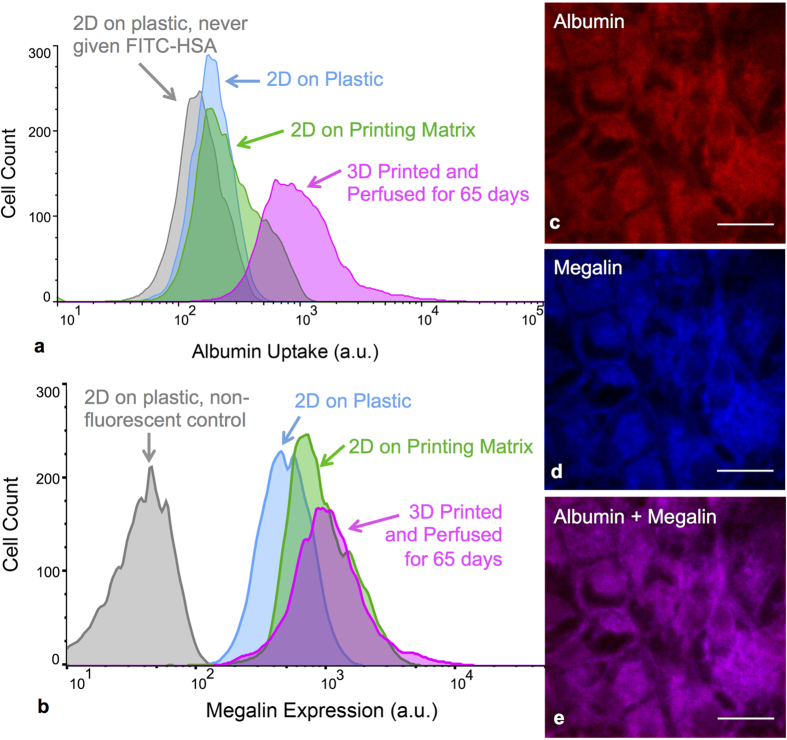
Improved functionality of printed and perfused 3D proximal tubules. **(a)** Albumin uptake assay in 3D proximal tubules. Flow cytometry data comparing the fluorescence intensity of PTECs fed FITC-labeled human serum albumin for 2 h under several conditions, including 2D controls on bare (blue) and ECM-coated (green) plastic dishes and in 3D PTs perfused for 65 days (magenta). (**b**) Flow cytometry data comparing the fluorescence intensity of megalin for the same PTEC samples as shown in (**a**,**c**) fluorescence image of the 3D PT constructs stained for FITC-labeled albumin (red), (**d**) megalin (blue), and (**e**) combined, scale bars = 20 μm.

Receptor-mediated endocytosis by PTEC cells is essential for body fluid homeostasis. Reabsorption of plasma proteins from the glomerular filtrate relies partially on the megalin-cubilin complex located in the brush border^35^,^36^,^37^ and can be modeled *in vitro* by monitoring albumin uptake by PTECs. We tested the ability of PTECs, grown either on perfused 3D PT constructs or 2D controls, to uptake FITC-labeled human serum albumin (HSA). After exposure to FITC-HSA for 2 h, PTECs are collected, stained for megalin expression, and analyzed by flow cytometry. The results for albumin uptake are provided in Fig. 4a. Large populations of cells in the 2D controls exhibit fluorescence intensity similar to the non-fluorescent control, whereas cells lining the perfused 3D PT constructs exhibit a significant increase in the FITC-HSA intensity. Results for megalin, one of the transporters for albumin, show that its expression is also highest in the 3D PT (Fig. 4b). Mean values for the fluorescence intensity of the populations analyzed by flow cytometry are provided in Table S1. Contrary to the 2D controls, we find that enhanced megalin expression is strongly correlated with superior albumin functional uptake in the perfused 3D PTs, suggesting that both their 3D architecture and perfusion improve epithelial function likely due to enhanced cell polarity and brush border (Fig. 3). Lastly, images of FITC-HSA (Fig. 4c), megalin (Fig. 4d), and the combination thereof (Fig. 4e) reveal an overlapping distribution of albumin and megalin in PTECs that line the 3D PT. Thus, our engineered 3D PT constructs exhibit superior albumin uptake function relative to either 2D control.

### Drug Toxicity Testing

**Figure 5:**
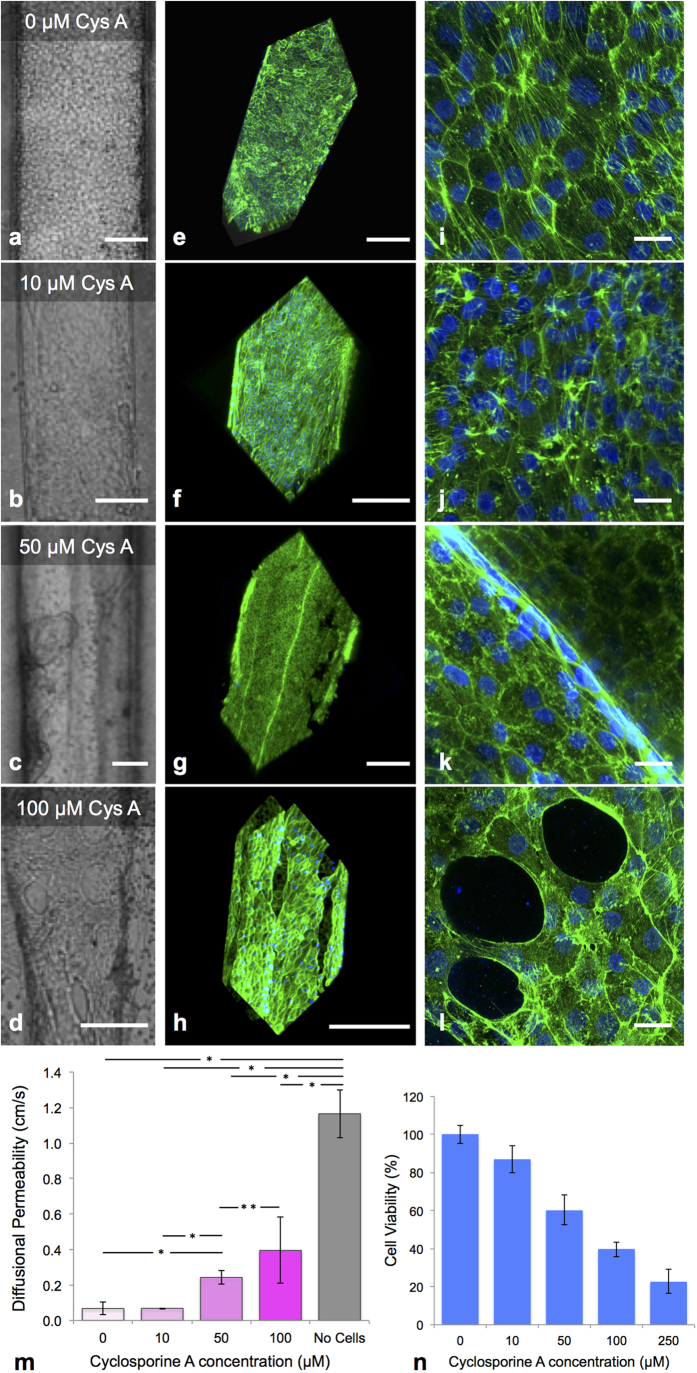
Cyclosporine A-induced cytotoxicity. **(a–d)** Brightfield images, (**e–h**) 3D renderings, and (**i–l**) high magnification images of printed and perfused 3D PTs dosed with varying concentrations of Cyclosporine A for 24 h, where actin (green) and nuclei (blue) are stained, scale bars = 200 μm (**a**–**h**) and scale bars = 20 μm (**i**–**l**), respectively, (**m**) Diffusional permeability measurements taken after dosing with Cyclosporine A, *p < 0.003, **p < 0.02, (**n**) Cell viability measured for the 2D control (on bare dish) after dosing with Cyclosporine A (all populations shown are statistically significantly different with a p < 0.005).

Cyclosporine A, a drug commonly given following transplant surgery to prevent rejection, is a known nephrotoxin that damages proximal tubule cells. To study its effect on the perfused 3D PT model, we exposed them to various concentrations of Cyclosporine A (CysA) and monitored alterations of cell morphology and cytoskeleton organization by immunostaining of actin filaments. Bright field images of the tubules (Fig. 5a–d) and corresponding 3D renderings of actin staining (Fig. 5e–l) reveal dose-dependent manifestation of CysA-induced damage. Minor breaks in cell-cell junctions (Fig. S8) and reorganization of actin (Fig. 5j) are observed at 10 μM CysA, whereas discrete areas devoid of cells are readily evident at 50 μM CysA (Fig. 5g,k) and those areas become more pronounced at 100 μM CysA (Fig. 5d,h,l and Movie S3). We also note that cell layers tighten and buckle at 50 μM and 100 μM CysA (Fig. 5g,k and Movies S2 and S3). Finally, we assessed CysA-induced disruption of the epithelial barrier function by quantifying the diffusional permeability of FITC-dextran (70 kDa) in treated tubules (Fig. S9). As shown in Fig. 5m, exposure to 50 and 100 μM CysA increases the epithelial barrier permeability by almost 4-fold and 6-fold, respectively. We also find that the respective cell viability of PTECs grown on 2D culture plastic dishes decreases by 40% and 60% after treatment with 50 and 100 μM CysA (Fig. 5n). Overall, these results indicate that the 3D PT constructs can be used to qualitatively (immunostaining) and quantitatively (diffusional permeability measurements) assess nephrotoxicity.

## Discussion

Recent advances in bioprinting enable the integration of pervasive and interconnected channels within engineered extracellular matrices^26^,^38^. We previously showed that these channels can be lined with endothelial cells and perfused to create tissues with embedded vasculature^25^,^26^. By combining bioprinting, 3D cell culture, and organ-on-chip methods, we demonstrate a customizable platform for fabricating perfusable, convoluted 3D proximal tubules on chip. Our ability to programmably define tubule size and geometry, including convolution, overcomes the limitations of pin pullout approaches that can only produce straight tubules in gels^31^. Our engineered ECM, which is based on enzymatic crosslinking of fibrinogen and gelatin^25^, promotes improved adhesion of PTECs relative to prior matrices^23^ allowing the cells to form a confluent layer that can be sustained for >60 days. This epithileum exhibits several morphological features and functional markers akin to native PTECs *in vivo*. Unlike kidney-on-a-chip devices based on cell monolayers^19^,^36^, our perfusable 3D PTs enable collection of hundreds of thousands of cells for analysis, far greater than that required (~10,000 cells) for accurate sampling via flow cytometry.

Our 3D PT models can be used to elucidate mechanisms of drug-induced tubule damage, including weakening of cell-cell junctions, cell ejection from the monolayer, and cell death. In the future, we will investigate the morphology and function of PTECs seeded within printed 3D tubules whose diameter (~60 μm) and curvature more closely mimics *in vivo* PTs to determine whether further improvements to the epithelium structure and function can be achieved. We also envision creating more complex 3D kidney models, in which both multiple tubules and vascular networks are patterned alongside one another to facilitate basal side access and studies of interactions between adjacent channels (Figs S1 and S10). By incorporating multiple cells types in the extratubular space (Fig. S5), we can introduce additional complexity required for studying cell-cell interactions. Ultimately, we plan to explore seeding and maturation of iPSC-derived renal progenitors in our perfusable 3D PT constructs.

In summary, we have reported the fabrication and characterization of 3D convoluted renal proximal tubules embedded within an extracellular matrix on customized perfusion chips. These perfusable 3D PTs promote the formation of a tissue-like epithelium with improved phenotypic and functional properties relative to the same cells grown on 2D controls. Our bioprinting method opens new avenues for creating 3D organs-on-a-chip that better recapitulate *in vivo* microenvironments, which could enable advances in drug screening, mechanistic drug studies, disease models, and ultimately, regenerative medicine.

## Methods

### Extracellular matrix preparation and rheology

The ECM is comprised of a network of gelatin and fibrin. To prepare the ECM components, a 15 wt/v% gelatin solution (Type A, 300 bloom from porcine skin, Sigma) is first produced by adding gelatin powder to a warm solution (70 °C) of DPBS (1X Dulbelco’s phosphate buffered saline without calcium and magnesium). The gelatin is allowed to fully dissolve by stirring for 12 h at 70 °C, and the pH is then adjusted to 7.5 using 1 M NaOH. The solution is sterile filtered and stored at 4 °C in aliquots for later usage in casting (<3 months). A fibrinogen solution (50 mg/mL) is produced by dissolving lyophilized bovine blood plasma protein (Millipore) at 37 °C in sterile DPBS without calcium and magnesium. The solution is held at 37 °C without agitation for at least 45 min to allow complete dissolution. The transglutaminase (TG) solution (60 mg/mL) is prepared by dissolving lyophilized powder (Moo Gloo) in DPBS without calcium and magnesium and gently mixing for 20 sec. The solution is then held at 37 °C for 20 min and sterile filtered before using. A CaCl_2_ stock solution (250 mM) is prepared by dissolving CaCl_2_ powder in DPBS without calcium and magnesium (Corning). To prepare stock solution of thrombin, lyophilized thrombin (Sigma Aldrich) is reconstituted at 500 U/mL using sterile DPBS and stored at −20 °C. Thrombin aliquots are thawed immediately prior to use.

A controlled stress rheometer (DHR-3, TA Instruments, New Castle, DE) with a 40 mm diameter, 2° cone and plate geometry is used for ink rheology measurements. The shear storage (*G*′) and loss (*G*″) moduli are measured at a frequency of 1 Hz and an oscillatory strain (γ) of 0.01. Time sweeps are conducted by rapidly placing a premixed ECM solution that contains thrombin onto the Peltier plate held at 37 °C.

### Ink formulations

Two inks are required for 3D bioprinting of perfusable PT models. One ink, which is used to create the perfusion chip gasket, is composed of a two-part silicone elastomer (SE 1700, DOW Chemical) with a 10:1 base to catalyst (by weight) that is homogenized using a centrifugal mixer for 2 min (2000 rpm, AE-310, Thinky Corp, Japan). The silicone ink is printed within 2 h of mixing with catalyst. This ink is loaded in a syringe (EFD Inc., East Providence, RI) and centrifuged to remove any air bubbles before printing at room temperature. The other ink, a fugitive ink used to print the tubule, is composed of 38 wt% Pluronic F127 (Sigma) and 100 U/mL thrombin in deionized, ultrafiltrated (DIUF) water. The fugitive ink is dyed pink through the addition of a Risk Reactor dye for visualization in Fig. 1 and Movie S1. To prepare this ink, a 40 wt% Pluronic F127 solution in water is homogenized using a Thinky mixer until the powder is fully dissolved, and subsequently stored at 4 °C. Prior to use, a 2000 U/mL thrombin solution is added to the fugitive (Pluronic) ink at a ratio of 1:20, and homogenized using a Thinky mixer. The fugitive ink is then loaded in a syringe (EFD Inc., East Providence, RI) at 4 °C and centrifuged to remove any air bubbles. Before printing, this ink is equilibrated at room temperature for at least 15 min.

### Bioprinting of perfusable 3D proximal tubule constructs

3D PT constructs are fabricated using a custom-designed, multimaterial 3D bioprinter equipped with four independently addressable printheads mounted onto a 3-axis, motion-controlled gantry with a build volume of 725 mm × 650 mm × 125 mm (AGB 10000, Aerotech Inc., Pittsburgh, PA USA). Inks are housed in separate syringe barrels to which nozzles of varying size (i.e., 50 μm–410 μm diameter) are attached via a luer-lock (EFD Inc., East Providence, RI, USA). Inks are extruded through deposition nozzles by applying air pressure (800 Ultra dispensing system, EFD Inc., East Providence, RI, USA), ranging from 10–90 psi, corresponding to print speeds between 1 mm/s and 5 cm/s. We first print the customized perfusion chip gasket by depositing the silicone ink through a tapered 410 μm nozzle onto 50 mm × 75 mm glass slides. The gasket design is created using a custom MATLAB script that generates G-code for a final gasket structure. After printing, the perfusion chip gasket is cured at 80 °C in an oven for >1 h and stored at room temperature prior to use.

Patterning 3D PTs within the perfusion chip requires a combination of casting the ECM and printing the fugitive ink. First, the ECM solution is created by combining 10 mg/mL fibrinogen, 7.5 wt% gelatin, 2.5 mM CaCl_2_ and 0.2 wt% TG. This solution is then equilibrated at 37 °C for 15–20 min before use to improve optical clarity of the ECM^25^. Next, the solution is rapidly mixed with thrombin at a ratio of 500:1, resulting in a final thrombin concentration of 1 U/mL. Within 2 min at 37 °C, polymerization of fibrinogen into fibrin gel ensues. For this reason, the ECM solution must be cast onto the base of the perfusion chip immediately after mixing with thrombin. The base ECM layer is then allowed to dry slightly under nitrogen, such that it forms a flat surface. The fugitive Pluronic F127 ink (with 100 U/mL thrombin) is printed on the base ECM layer in the form of a convoluted filmament (tubule) using a tapered 200 μm nozzle. A custom Python script (MeCode) is used to specify the toolpath in G-code. Directly after fugitive ink printing, metal hollow perfusion pins interfaced through the silicone gasket are brought into contact with the printed ink. A top layer of ECM is then formed by casting the ECM solution over the printed tubule, as described above, to within 1–2 mm of the height of the gasket walls. If cells, such as HNDFs, are incorporated in the ECM (Fig. S5), they are mixed in directly after the equilibration period, prior to thrombin mixing and subsequent casting. After the top ECM layer is cast, the construct is covered with a glass slide to prevent evaporation or contamination and is held at 37 °C for 1 h to allow fibrin polymerization to terminate and TG to crosslink the network. The construct is then cooled to 4 °C for 15–20 min to liquefy the printed fugitive ink, which is flushed out of the device using cold cell media, leaving behind open conduits that serve as the desired tubular network embedded within the ECM with or without cells in the extratubular ECM space.

Using this method, we also produced 3D architectures in a layer-by-layer build sequence. For example, each individual layer of the three-layer structure shown in Fig. S10 has been constructed using a modified printing protocol that incorporates the materials and methods previously discussed. After printing the first tubules with fugitive ink, a layer of ECM is cast over the print and permitted 20 min to gel at 37 °C before the next proximal tubule layer is printed with fugitive ink on top of the recently gelled layer. This successive construction introduces 3D geometry and permits successful evacuation of all channels independently after construction. Aqueous-based risk reactor dyes are perfused through the channels and excited with UV light for visualization.

To complete the 3D tissue chip assembly process, each PT construct is placed onto a machined stainless steel base and a thick acrylic lid is placed on top. The lid and base are clamped together by four screws, forming a seal around the printed silicone gasket. Next, sterile two-stop peristaltic tubing (PharMed BPT, 0.25 mm internal diameter) is filled with media and connected to the outlet of a sterile filter that is attached to a 10 ml syringe barrel (EFD Nordson), which serves as a media reservoir. PTEC media (designed for growth, so ATCC formulation plus 1% FBS, 1% aprotinin, and 1% anti-anti) that has been equilibrating for >3 h in an incubator at 37 °C, 5% CO_2_ is added to the media reservoir, and tubing from the reservoir is connected to the outlet of the chip (metal hollow perfusion pin). A syringe is then used to exert slight pressure on the media in the barrel, forcing it to enter and completely fill the attached tubing. Filling the tubing with media prior to connecting it to the circuit prevents the introduction of air bubbles into the system. To complete the perfusion circuit, silicone tubing from the reservoir is connected to the inlet metal perfusion pin on the chip. Hose pinch-off clamps are added at the inlet and outlet of the perfusion chip to prevent uncontrolled flow when disconnected from the peristaltic pump, which can damage the epithelium or permit air bubbles to enter the system. The media reservoir is equilibrated with atmospheric conditions in the incubator at all times by means of a sterile filter on top of the media reservoir.

### Cell Culture

Human immortalized PTECs (RPTEC/TERT1, ATCC CRL-4031) are cultured per ATCC’s instructions and are used for all PT model studies up to passage 20. For gene expression analysis, human primary RPTEC (Cell Science), immortalized PTECs (RPTEC-TERT1, Evercyte) and A498 (ATCC HTB-44) renal cancer cells are used and cultured per supplier’s instructions. Human neonatal dermal fibroblasts (HNDF), GFP expressing (Angio-Proteomie) are cultured per supplier’s instructions and used up to passage 15.

### Gene expression analysis

Human primary RPTEC (Cell Science), immortalized RPTEC-TERT1 (Evercyte) and A498 (ATCC HTB-44) renal cancer cells are grown in 96-well plates according to supplier’s instructions and collected at Day 3 post-confluency by replacing culture medium with 100 μl/well of 1x RNA lysis mixture (QuantiGene Sample Processing Kit, QS0101). Then 40 μl of lysate is mixed with an mRNA-capture magnetic bead set (Panomics QuantiGene Plex Set 12631, catalog number 312631), incubated overnight, processed for branched DNA amplification, and analyzed according to the manufacturer’s instructions (Panomics QuantiGene Plex Assay kit, QP1015). The PPIB probe is used as a housekeeping gene for normalization. Fluorescence Intensity (FI) data are presented as average and standard deviation of 3 biological replicates.

### Cytokine analysis of media perfusate

Media perfusate is collected from a tubule over a period of 25 days post cell seeding and stored at −80 °C prior to analysis. For cytokine profiling, supernatants are thawed on ice, diluted 2x in sample dilution buffer (BioRad catalog #M60-009RDPD) and analyzed by Luminex technology-based ELISA using the Bio-Plex Pro™ Human Chemokine IL-6 (Set #171BK29MR2), IL-8 (Set #171-BK31MR2) and MCP-1 (Set #171-BK36MR2) and the Bio-Plex 200 Systems (BioRad) according to the manufacturer’s instructions. Data are reported as average cytokine concentrations and standard deviations of technical triplicates.

### Epithelialization and longitudinal culture

Each 3D PT construct is perfused for several hours with PTEC media in the incubator prior to cell loading/seeding. PTECs (PTEC/TERT1, ATCC) are trypsinized from their culture dish and concentrated in media to ~2 × 10^7 ^cells/mL. The cell suspension is then loaded into the perfusion chip through the outlet (Fig. S3b,c). The loaded construct is placed laterally in the incubator for several hours and flipped 180° over the course of multiple half-hour intervals to allow for uniform seeding of the tubule walls, then incubated in the tubule with no flow overnight. The next day, non-adherent cells are flushed out of the tubule under flow by gravity. Perfusion of fresh media is then started and the remaining cells begin to cluster and then grow from those colonies (Fig. S3f) until they reach confluency at around 3 weeks post seeding (Fig. S3k). During the growth phase, PTECs are fed PTEC media prepared per ATCC guidelines plus 1% aprotinin (EMD Millipore, used to slow down the degradation of the ECM), 1% fetal bovine serum (FBS), and 1% antibiotic-antimycotic (Gibco). After maturation, FBS is removed, and PTECs pack into a tight epithelial monolayer (Movie S2). At Day 1 post-seeding, the PTECs are exposed to continuous, unidirectional flow at 1 μl/min, equating to shear stresses that vary between 0.1 and 0.5 dynes/cm^2^ depending on the tubule cross section. Media is fed via a peristaltic pump in a closed loop circuit and changed every 2 days.

### Albumin uptake study

Albumin uptake is assessed for the printed 3D PT models as well as 2D controls. The first control consists of PTECs grown on tissue culture plastic, while the second control consists of PTECs grown on our ECM. In each case, PTECs are grown to confluency and allowed to mature in serum free media. Human serum albumin conjugated with FITC (HSA-FITC, Abcam ab8030) is suspended in PTEC media at 50 μg/mL. All samples are incubated with HSA-FITC in their media for 2 h (in the case of perfusion, it is perfused through the open lumen). After exposure, all samples are washed with 3x volume and then trypsinized with 10x trypsin to collect the individual cells. Cells are fixed and counterstained with primary and secondary antibodies for megalin (Table S2 lists the specific antibodies used). Cells from those samples, and naked cells, are analyzed by flow cytometry (BD LSR Fortessa) and data is collected from n = 10,000 cells per sample. To obtain images of HSA-FITC and megalin in PTECs, samples are fixed in place with formalin instead of being trysinized after the wash step. Those samples are counterstained for megalin and imaged using confocal microscopy (Zeiss LSM710).

### Cyclosporine A testing

The effect of CysA on both 2D controls and bioprinted 3D PTs is explored. In 2D, cells are seeded in a 96-well format on tissue culture plastic and grown to confluency. They are fed media per ATCC’s guidelines. CysA (Sigma-Aldrich, SML1018) is suspended in their media at various concentrations and incubated with cells for 24 h. A viability assay using (3-(4,5-dimethylthiazol-2-yl)-5-(3-carboxymethoxyphenyl)-2-(4-sulfophenyl)-2H-tetrazolium) in the presence of phenazine methosulfate (MTS) is run at the 24 h mark post exposure. This assay is completed on PTECs at early confluency, by giving CysA to the cells on the day they reached confluency, as well as late confluency, by giving CysA several days after they reached confluency. Notably, the toxicity results are similar for each case (Fig. 5n). For 3D PTs, CysA is fed at various concentrations through the open lumen of mature tubules after reaching confluency (at ~3 week mark), where no serum is included in the media for a minimum of 10 days. At the 24 h mark post CysA exposure, a FITC-dextran leak test (described below) is performed to assess and quantify perturbations to the barrier function of PTECs. Directly following, the PT is fixed using 10% buffered formalin for 1 h and counterstained for actin and DAPI (Table S2 lists the specific stains used).

### Diffusional permeability measurements

To assess barrier function of the epithelium in 3D, diffusional permeability is quantified by perfusing PTEC media in the open lumen containing 25 μg/mL FITC-conjugated 70 kDa dextran (FITC-Dex, Sigma product 46945) at a rate of 15 μL/min for 3 min and 1 μL/min thereafter for ~30–45 min. The entire test is performed under live cell imaging with both the tubule and the surrounding ECM in the field of view (Fig. S9). The diffusion pattern of FITC-Dex is detected using a wide-field fluorescent microscope (Zeiss Axiovert 40 CFL). Fluorescence images are captured before perfusion and every 3 to 5 min over a 30–45 min period. Diffusional permeability of FITC-Dex is calculated by quantifying changes in fluorescence intensity over time using the following equation^34^;


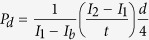


*P*_*d*_ is the diffusional permeability coefficient, *I*_*1*_ is the average intensity at an initial time point, *I*_*2*_ is an average intensity at *t* ~ 30–45 min, *I*_*b*_ is background intensity (image taken before perfusion of FITC-Dex), and *d* is the diameter of the channel. Other researchers have reported that PTECs can resorb dextran^39^, which would lead to slightly higher values for the measured diffusional permeability.

We also investigated the barrier properties of our epithelial lined tubules using a low molecular weight compound, inulin (4.5 kDa) that is neither resorbed nor secreted *in vivo* by PTECs using the same method described above. Specifically, inulin-FITC (Sigma product F3272) is dissolved in warmed PTEC media at 100 μg/mL and perfused in the open lumen at a rate of 20 μL/min for 3 min and 1.5 μL/min thereafter for ~15 min. The entire test is performed under live cell imaging with both the tubule and the surrounding ECM in the field of view (Fig. S7). The diffusion pattern of FITC-inulin is detected using a wide-field fluorescent microscope (Leica). Fluorescence images are captured with a gated light source and motion controlled stage before perfusion and every 3 to 5 min over the 15 min period to collect technical triplicate measurements.

### Electron microscopy

For transmission electron microscopy (TEM), PTECs in 2D or 3D architectures or healthy human kidney tissue obtained from a standard biopsy prior to transplant are fixed using 2.5% glutaraldehyde, 1.25% paraformaldehyde, and 0.03% picric acid in 0.1 M sodium cacodylate buffer (pH 7.4) for a minimum of several hours. Small samples (1 mm × 1 mm) are removed and washed in 0.1 M cacodylate buffer and bathed in 1% osmiumtetroxide (OsO_4_) (EMS) and 1.5% potassiumferrocyanide (KFeCN_6_) (Sigma) for 1 h, washed in water 3x and incubated in 1% aqueous uranyl acetate (EMS) for 1 h followed by 2 washes in water and subsequent dehydration in varying grades of alcohol (10 min each; 50%, 70%, 90%, 2 × 10 min 100%). The samples are then put in propyleneoxide (EMS) for 1 h and incubated overnight in a 1:1 mixture of propyleneoxide and TAAB Epon (Marivac Canada Inc. St. Laurent, Canada). The following day the samples are embedded in TAAB Epon and polymerized at 60 °C for 48 h. Ultrathin sections (about 60 nm) are cut on a Reichert Ultracut-S microtome, placed on copper grids stained with lead citrate and examined in a JEOL 1200EX Transmission electron microscope and images are recorded with an AMT 2k CCD camera. Image analysis is performed using ImageJ software.

For scanning electron microscopy (SEM), perfused PTECs in 3D are fixed using 10% buffered formalin for 1 h. The samples are thinly sliced (~1 mm thick) to expose cells circumscribing the open lumen. The fixative is washed away using PBSx2 and subsequent dehydration in varying grades of ethanol (20 min each; 30%, 50%, 70%, 90%, 3 × 20 min 100%). The samples are then placed in 50% ethanol and 50% hexamethyldisilazane (HMDS) for 30 min followed by 100% HMDS 3 × 30 min. All steps are performed in a closed and sealed glass container. After the final washing with HMDS, the samples are removed and placed in an open container under N_2_ in the fume hood to dry. Dried samples are mounted to aluminum pin mounts using conductive carbon tape, sputter coated with gold, and imaged with a Tescan Vega SEM.

### Immunostaining

Immunostaining followed by confocal microscopy is used to assess the cellular localization of proteins in 2D and 3D PTEC models. Prior to immunostaining, each construct is washed with PBS and then fixed for 20 min to 1 h using 10% buffered formalin. The fixative is removed using several washes in PBS for several hours and then blocked overnight using 1 wt% bovine serum albumin (BSA) in PBS. Primary antibodies to the cell protein or biomarker of interest are incubated with the constructs for 1 day at the dilutions listed in Table S2 in a solution of 0.5 wt% BSA and 0.125 wt% Triton X-100. Removal of unbound primary antibodies is accomplished using a wash step against a solution of PBS or 0.5 wt% BSA and 0.125 wt% Triton X-100 in PBS for 1 day. Secondary antibodies are incubated with the constructs for 1 day at the dilutions listed in Table S2 in a solution of 0.5 wt% BSA and 0.125 wt% Triton X-100 in PBS. Samples are counter-stained with NucBlue or ActinGreen for 2 h and then washed for 1 day in PBS prior to imaging.

### Image rendering and analysis

Phase contract microscopy is performed using an inverted Leica DM IL scope with objectives ranging from 1.25X to 40X. Confocal microscopy is performed using an upright Zeiss LSM 710 with water immersion objectives ranging from 5X to 40X employing spectral lasers at 405, 488, 514, 561, and 633 nm wavelengths. Image reconstructions of z-stacks are performed in ImageJ using the z-projection function with the maximum pixel intensity setting. Any increases in brightness are performed uniformly across an entire z-projected image. 3D image reconstructions and rotating movies (Movie S3) are performed using Imaris software. The new CytoSMART (Lonza) in incubator system is used to capture time-lapse imaging (Movie S2). Image analysis for quantification of diffusional permeability is performed using custom MATLAB scripts employing previously reported methods^34^. TEM image analysis is performed using ImageJ software to measure cell height (*n* ≥ 50), microvilli density (*n* ≥ 25), and microvilli length (*n* ≥ 150) over at least 3 independent samples for each condition.

### Statistical analysis

Data are expressed as means ± standard deviation. Statistical analysis is performed using MATLAB and statistical significance is determined at a value of p < 0.05 as determined by an ANOVA using Tukey’s multiple pairwise comparison test. Different significance levels (p values) are indicated with asterisks and specific p values are provided in each figure legend.

## Additional Information

**How to cite this article**: Homan, K. A. *et al*. Bioprinting of 3D Convoluted Renal Proximal Tubules on Perfusable Chips. *Sci. Rep.* **6**, 34845; doi: 10.1038/srep34845 (2016).

## Supplementary Material

Supplementary Movie 1

Supplementary Movie 2

Supplementary Movie 3

Supplementary Information
