# Supplementary material for: Bioprinting of 3D Convoluted Renal Proximal Tubules on Perfusable Chips
Source: Sci Rep. 2016 Oct 11;6:34845. doi: 10.1038/srep34845 (PMC5057112; doi:10.1038/srep34845)
Supplement: Supplementary Information [file srep34845-s4.pdf]

Bioprinting of 3D Convoluted Renal Proximal Tubules on Perfusable Chips

Kimberly A. Homan[†1,2], David B. Kolesky[†1], Mark A. Skylar-Scott[1], Jessica Herrmann[1], Humphrey Obuobi[1], Annie Moisan[†*2], and Jennifer A. Lewis[†*1]

[†] These authors contributed equally to this work
[1]School of Engineering and Applied Sciences, Wyss Institute for Biologically Inspired Engineering, Harvard University, Cambridge, Massachusetts, United States of America.
[2]Pharmaceutical Sciences, Roche Pharma Research and Early Development, Roche Innovation Center, Basel, Switzerland.
*Corresponding author's email: annie.moisan@roche.com and jalewis@seas.harvard.edu

Supplementary Information

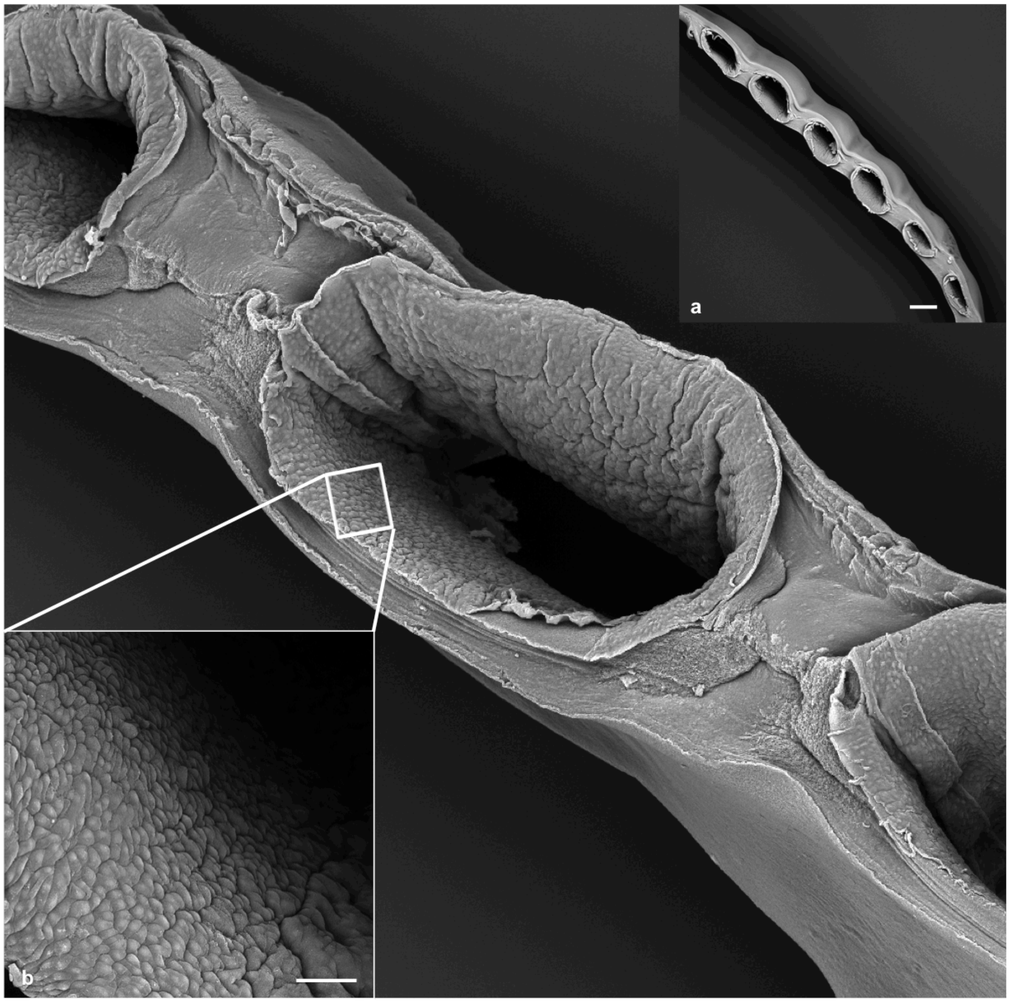

Figure S1. Multiplexed 3D proximal tubules. (a) SEM image of 6 PTs printed adjacent to one another, scale bar = 500 μm. [Note: The image is acquired on a thin dried slice cut from the printed sample.], (**b**) High magnification image taken inside the larger 3D PT shown in the background, scale bar = 50 μm. As shown here, multiple PTs can be printed in parallel and lined with PTEC cells that grow to confluency.

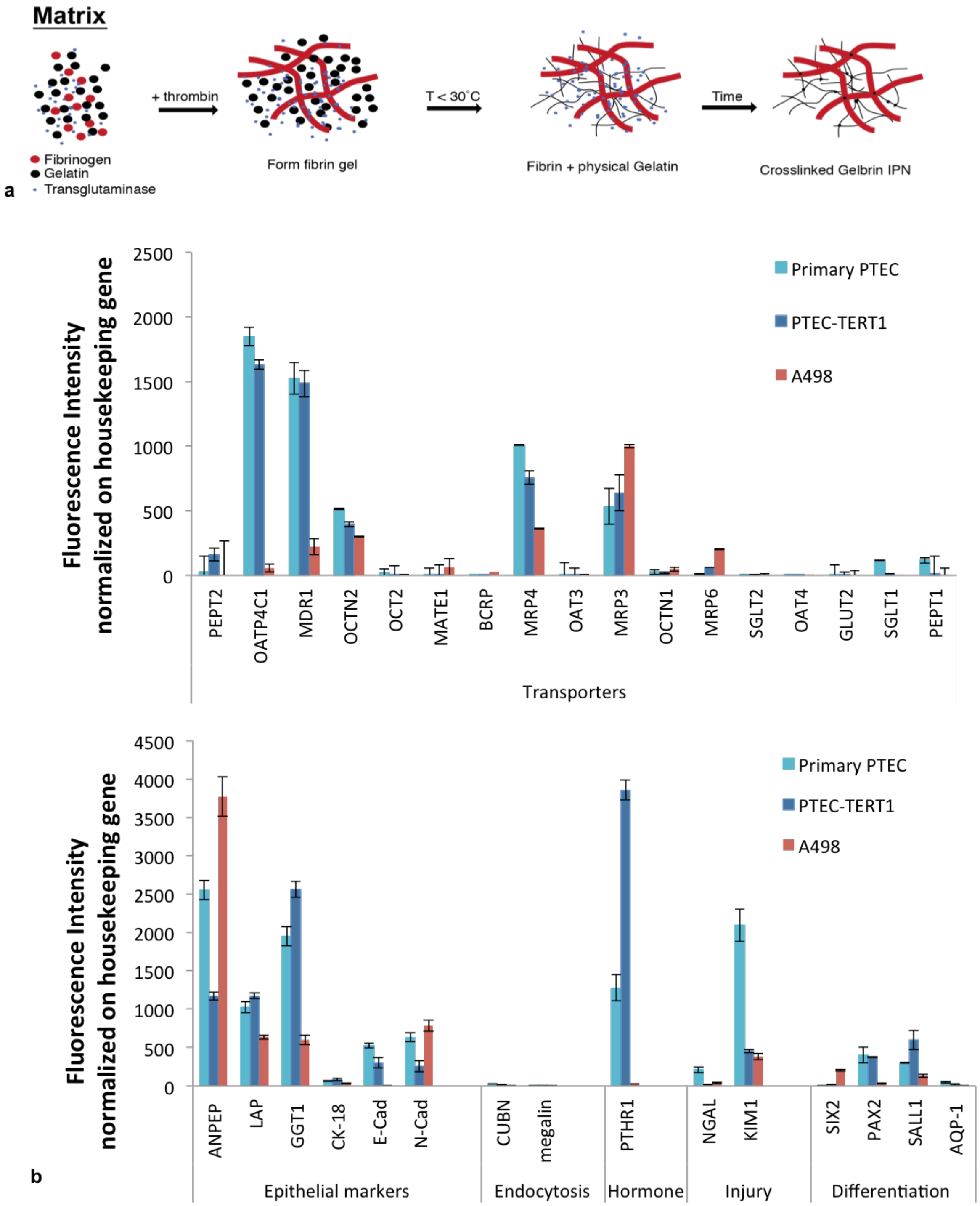

Figure S2. Engineered extracellular matrix (ECM) and gene expression profiles for various PTEC lines. (a) Schematic representation of the ECM constituents and their gelation and cross-linking as a function of different stimuli, (**b**) relative mRNA levels of 33 selected genes related to renal epithelial function, transport, endocytosis, hormone response, injury response, and cell differentiation for three cell lines (primary renal PTEC, PTEC-TERT1, and the A498 cancer renal cell line). PTEC-TERT1 cells are transcriptionally similar to primary PTEC and different from the A498 renal cancer epithelial cell line.

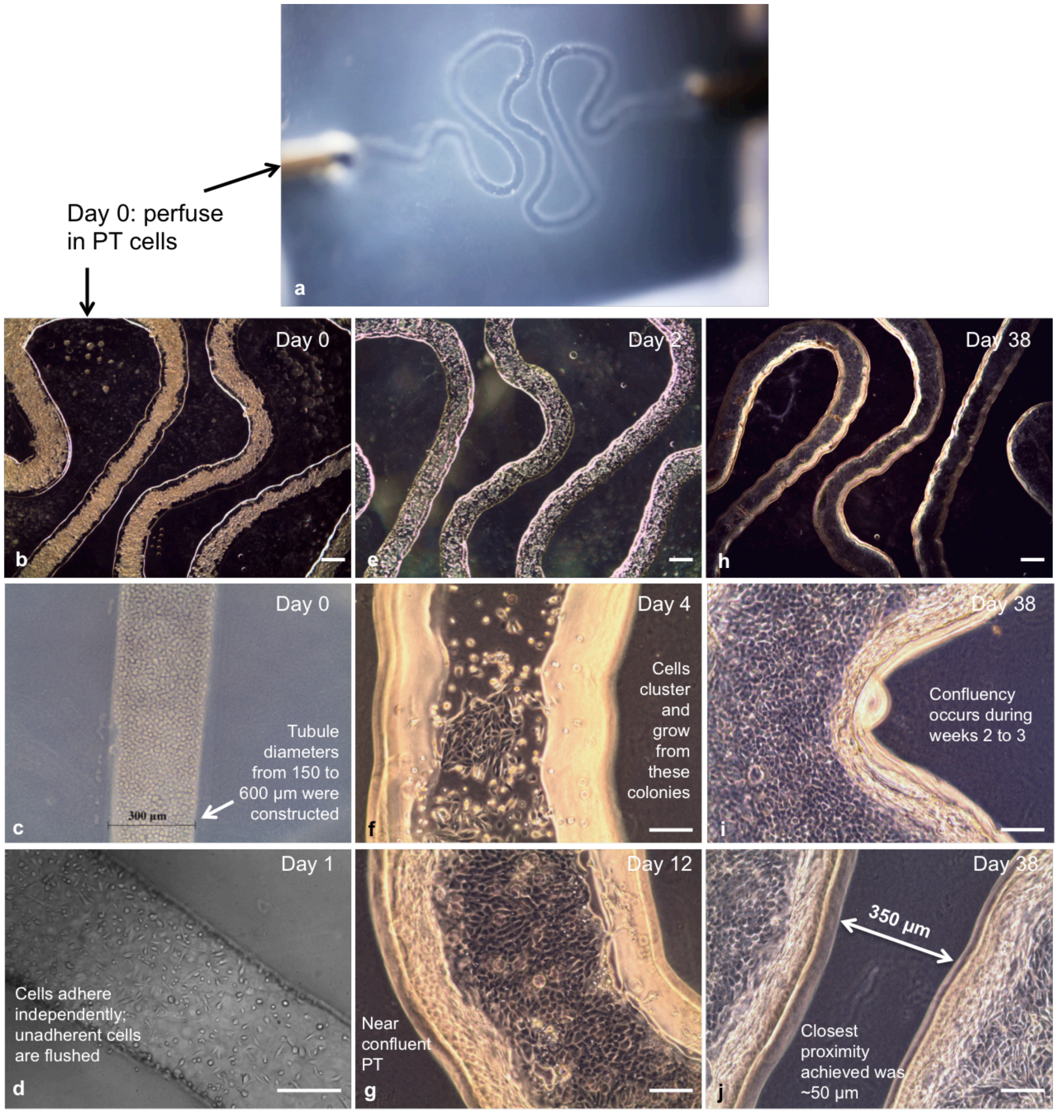

Timeline

k

Day -1	Day 0	Days 12-14	Days 22-24	> Day 60
PT construct made and perfused overnight	PTECs loaded by perfusion in the open lumen	PTECs near confluency in the PT and FBS is removed from the media	Cells are confluent and stable; ready for an assay or test	PT can be maintained longitudinally out to > 60 days

Figure S3. 3D proximal tubule maturation process. (a) A photo of a mature (fully confluent) tubule, (**b**) PTEC loading at Day 0, scale bar = 500 µm, (**c**) higher magnification view of PTEC loading, scale bar = 300 µm, (**d**) PTECs adhering to the tubule at Day 1 after non-adherent cells are flushed away, scale bar = 200 µm, (**e**) low magnification view of PTECs growing into the tubule at Day 2, scale bar = 500 µm, (**f**) image at Day 4 where cells grow from colonies or clusters, scale bar = 100 µm, (**g**) image at Day 4 where cells are near confluency, scale bar = 100 µm, (**h**) image of a mature tubule at Day 38, scale bar = 500 µm, (**i**) higher magnification view of the confluent tubule at Day 38, scale bar = 100 µm, (**j**) image of the tubule, which approaches within 350 µm of itself due to its convoluted architecture, scale bar = 100 µm, (**k**) timeline of construction and maturation of the PT model.

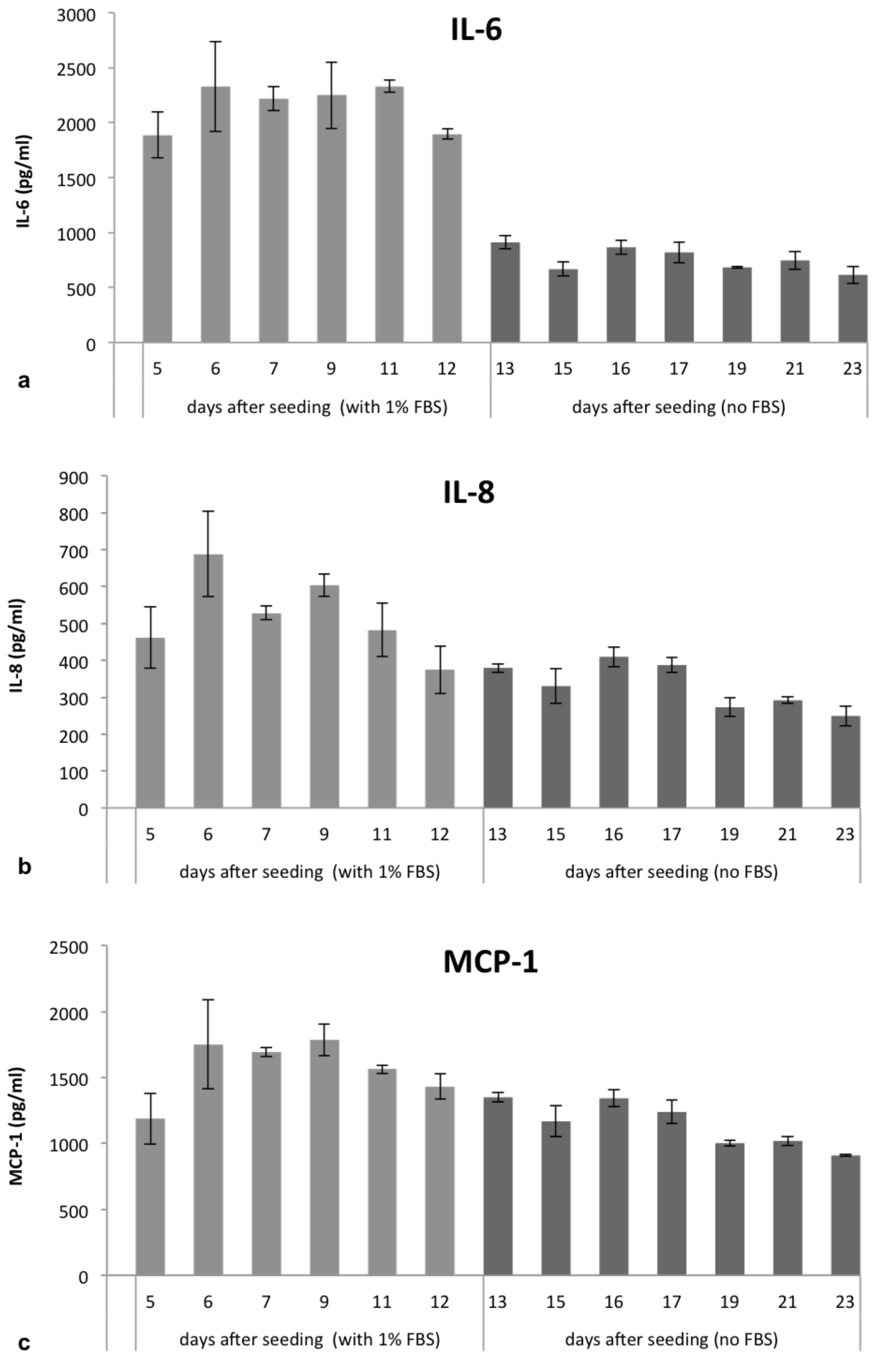

Figure S4. 3D proximal tubule perfusate analysis. The relative concentration of **(a)** IL-6, **(b)** IL-8, and **(c)** MCP-1, shed in the media perfusing through the tubule with time. The light grey bars represent the growth phase of the tubule. At Day 12, the tubule is near confluency, FBS is removed from the media, and the profile of the confluent tubule is shown in dark grey bars. Note that once confluency is reached and FBS is removed, cytokine levels stabilize.

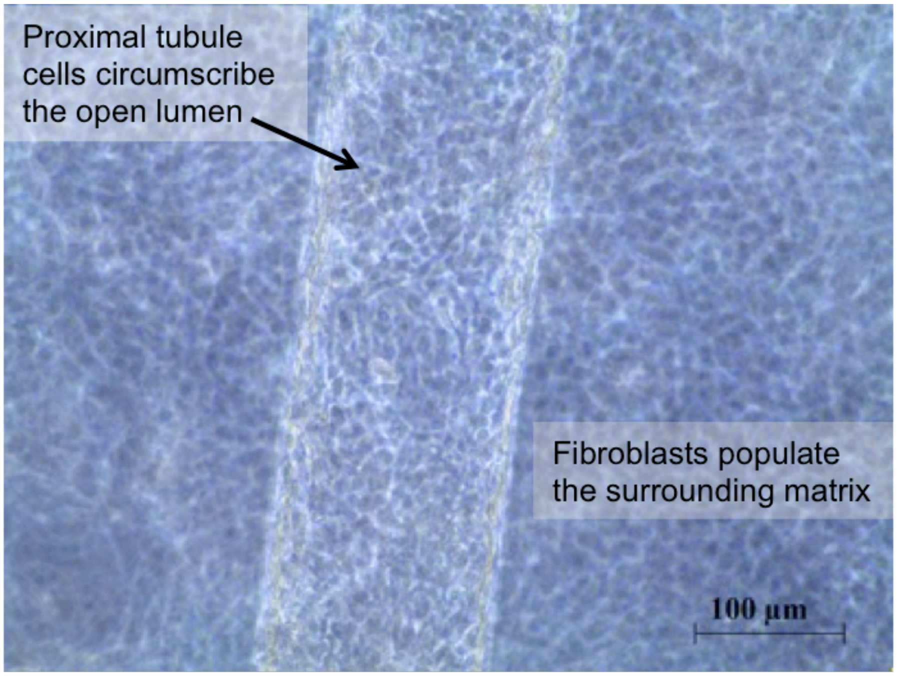

Figure S5. 3D proximal tubule lined with PTEC cells and embedded in a fibroblast-laden extracellular matrix. Phase contrast image of a 3D PT grown to a confluent epithelium, in which fibroblasts thrive in the surrounding ECM, scale bar = 100 µm.

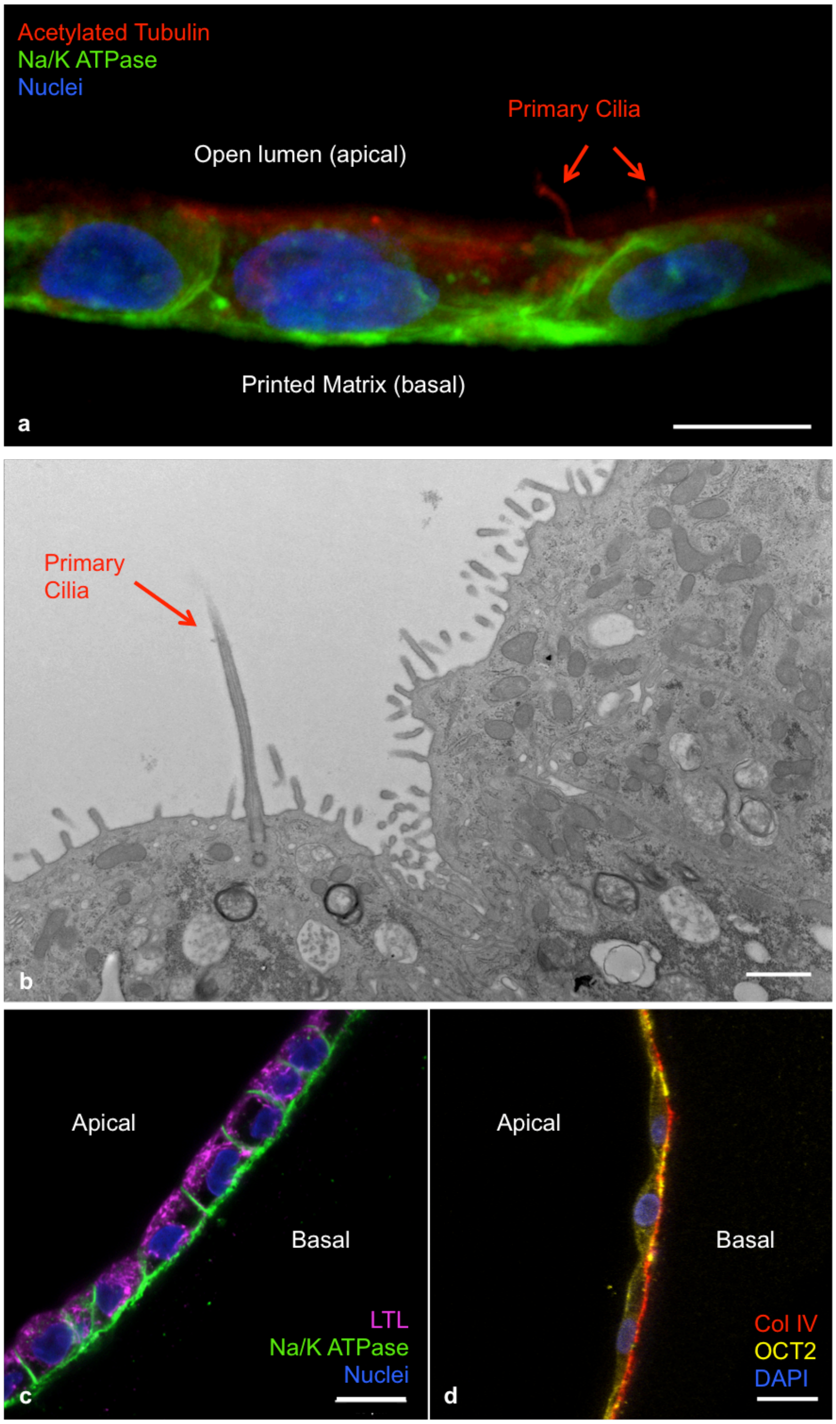

Figure S6. PTEC characterization within printed and perfused 3D proximal tubules. (a) 3D reconstruction of PTECs stained for Na^+/K^+ ATPase (green) and acetylated tubulin (red) where basal-lateral expression of Na^+/K^+ ATPase is apparent and two primary cilia are visible on the apical side, scale bar = 10 µm and (**b**) TEM image of primary cilia, scale bar = 1 µm. (**c**) Cross-section of the tubule showing apical expression of LTL (magenta) and basal expression of Na/K ATPase (green), scale bar = 15 µm, (**d**) Cross-section of the tubule showing basal expression of OCT2 (yellow) and collagen IV (red), scale bar = 15 µm.

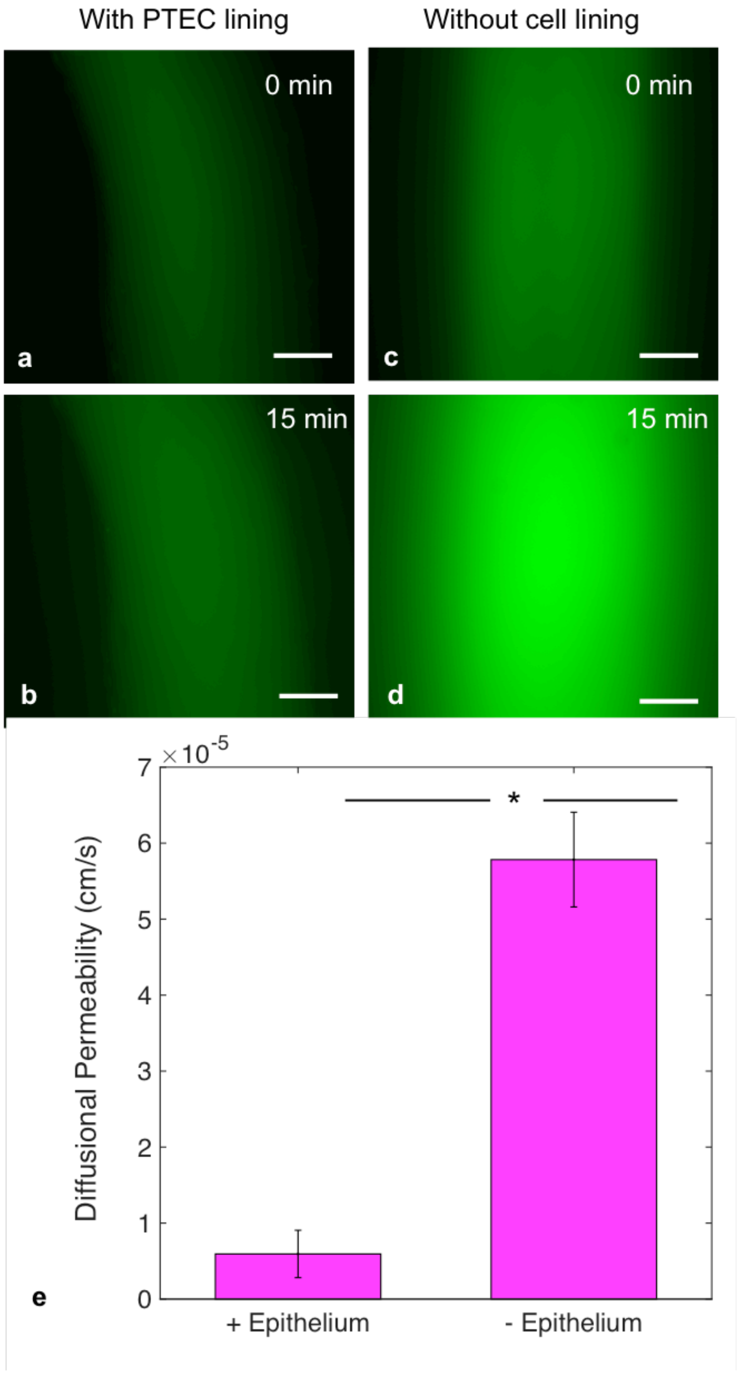

Figure S7. Diffusional permeability measurements. FITC-labeled inulin (4.5 kDa) suspended in cell media is perfused through the 3D PT lined with confluent PTECs and fluorescent images are captured at varying times: **(a)** t = 0 min and (**b**) t = 15 min for cell lined channels, and (**c, d**) t = 0 min and 15 min, respectively, for control samples composed of a bare 3D PT (without PTECs), in which the FITC-labeled inulin diffuses much faster into the surrounding ECM, scale bars = 100 µm. (**e**) Measured diffusional permeability of 3D PT channels with and without proximal tubule epithelium, *p > 0.001.

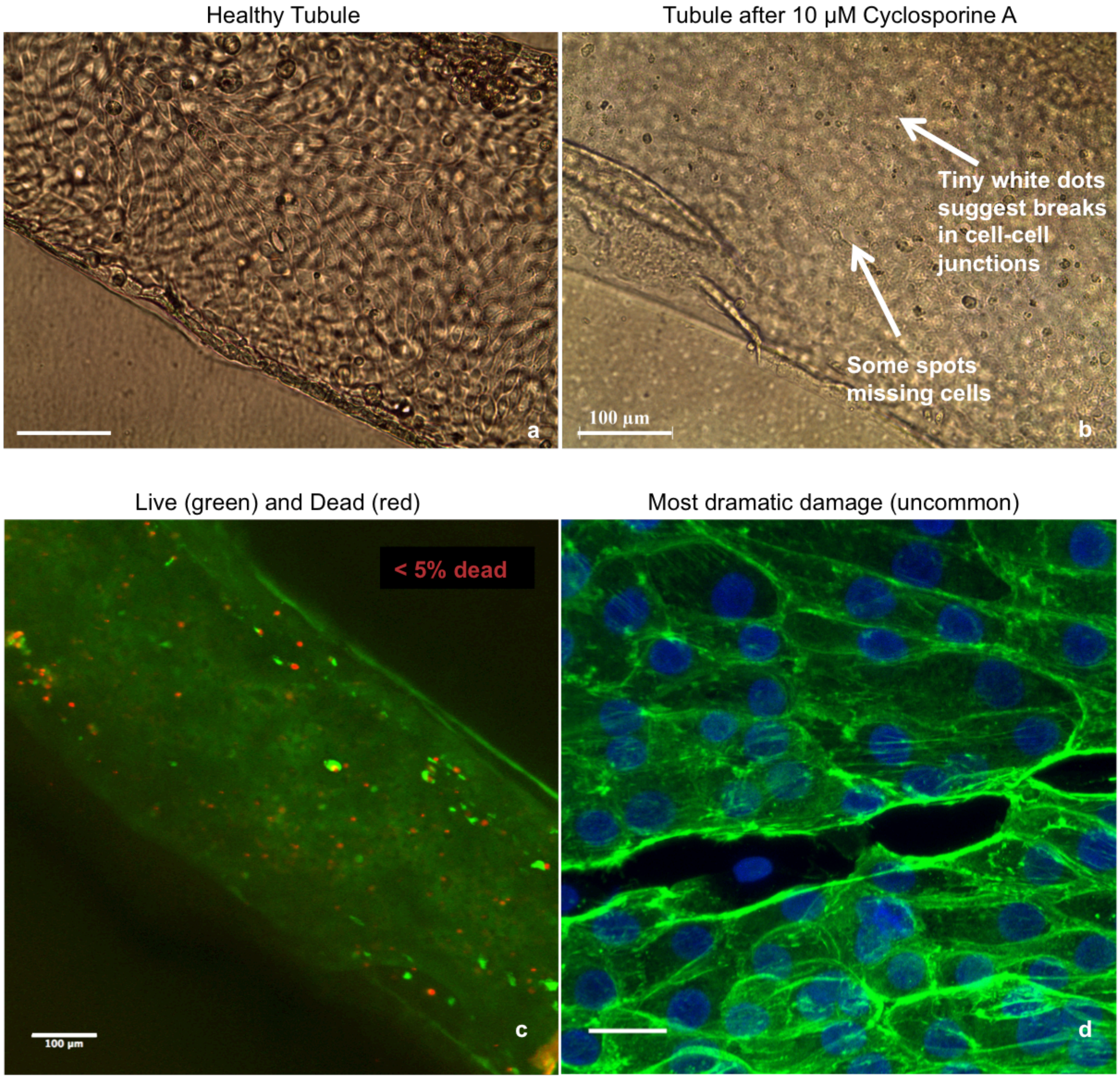

Figure S8. Observed damage for printed and perfused 3D proximal tubules dosed with 10 µM cyclosporine A. (a) Brightfield image of a healthy proximal tubule at 4 weeks, scale bar = 100 µm, (**b**) brightfield image of a tubule after 24 h of cyclosporine A exposure, scale bar = 100 µm, (**c**) live (green) and dead (red) staining of the tubule at 24 h after cyclosporine A exposure showing that < 5% of the total cells are dead, scale bar = 100 µm, (**d**) high magnification image showing the most dramatic, but quite uncommon, damage observed under these conditions, where actin (green) and nuclei (blue) are stained, scale bar = 20 µm.

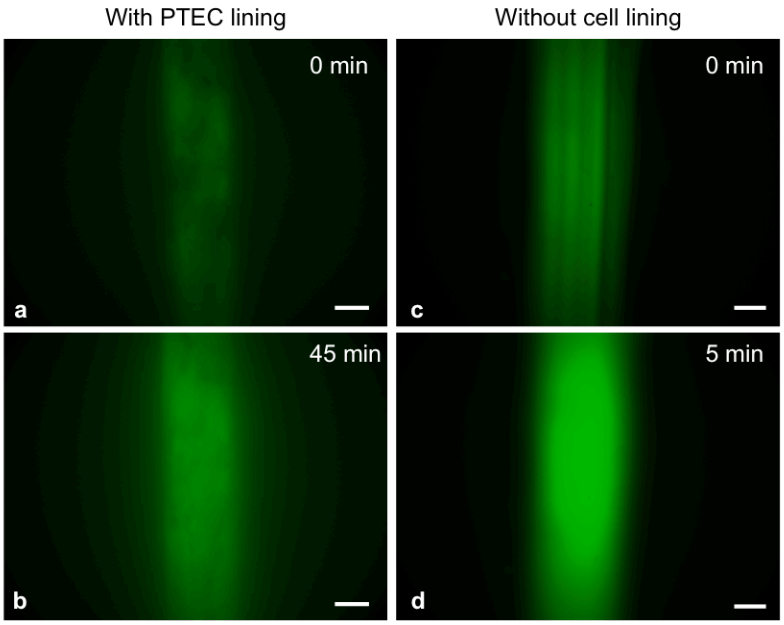

Figure S9. Diffusional permeability measurements for the Cyclosporine A study. FITC-labeled dextran (70 kDa) solution is perfused through the 3D PT lined with confluent PTECs and fluorescent images are captured at varying times: **(a)** t = 0 min and (**b**) t = 45 min for cell lined channels, and (**c, d**) t = 0 min and 5 min, respectively, for control samples composed of a bare 3D PT (without PTECs), in which the FITC-labeled dextran diffuses much faster into the surrounding ECM, scale bars = 200 μm.

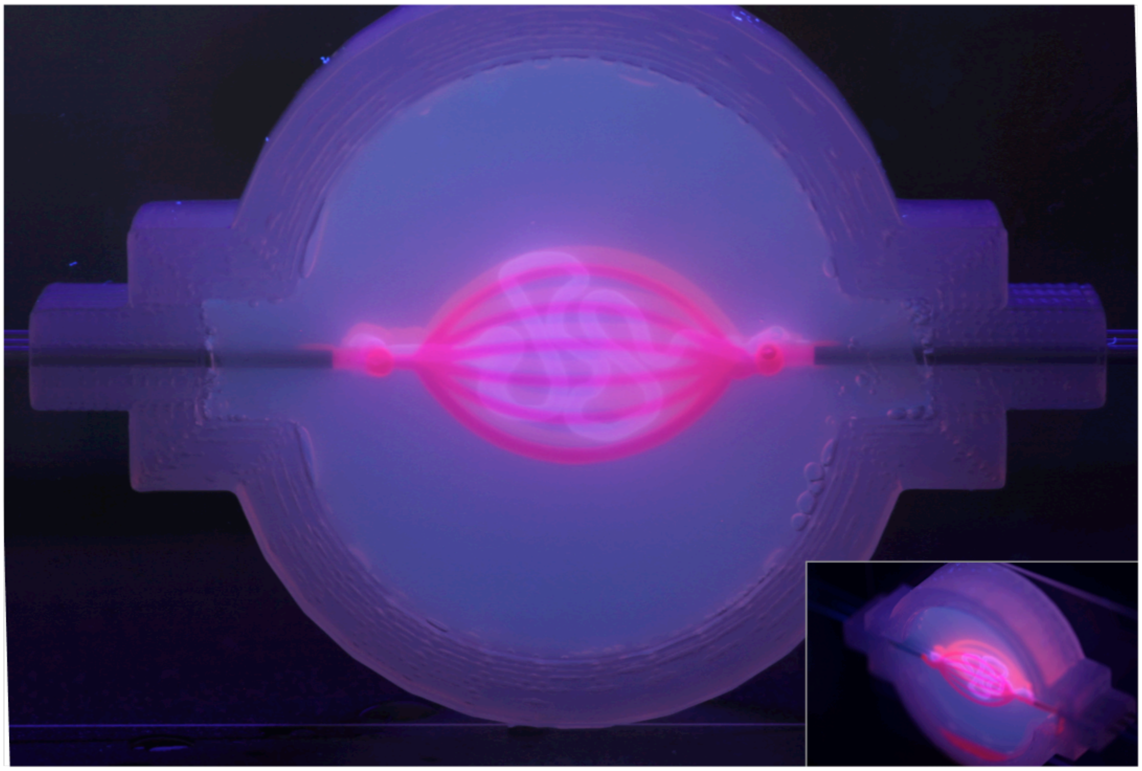

Figure S10. A photograph of our PT model constructed with 3 layers of independently addressable perfusable tubes. The inset shows the 3 pins connected to 3 separate tubes perfused with fluorescent dyes. This multi-layer model is a demonstration showing how bioprinting can be combined with microfluidics to interface vascular layers and proximal tubules in 3D.

Table S1. Albumin uptake for PTEC cells. Mean values of the data shown in **Fig. 4** for each population of cells.

Mean Intensity	Albumin	Megalin
2D on Plastic	201	571
2D on Printing Matrix	310	1127
3D Printed (Perfused)	1452	1670

Table S2. Immunostaining reagents.

Antibody or stain:	Source	Catalog #	Host Species & Reactivity	Concentration
Megalin	abcam	ab76969	Rabbit anti-human	1:300
AQP1	Santa Cruz	SC25287	Mouse anti-human	1:300
Na/K ATPase	abcam	ab76020	Rabbit anti-human	1:400
Acetylated alpha tubulin	abcam	ab24610	Mouse anti-human	1:300
Antibody to laminin	abcam	ab11575	Rabbit anti-human	1:250
K Cadherin	abcam	ab133632	Rabbit anti-human	1:200
OCT2	abcam	ab170871	Rabbit anti-human	1:200
LTL	Vector Lab	B-1325	N/A	1:200
ActinGreen	Life Technologies	R37110	N/A	2 drops per mL
NucBlue	Life Technologies	R37605	N/A	2 drops per mL

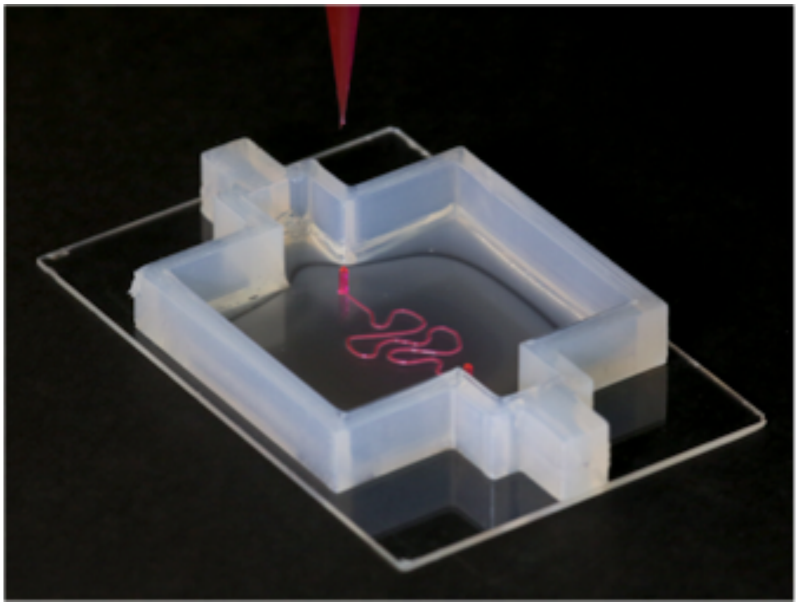

Movie S1. Fabrication of a 3D convoluted proximal tubule embedded within an extracellular matrix on a perfusion chip.

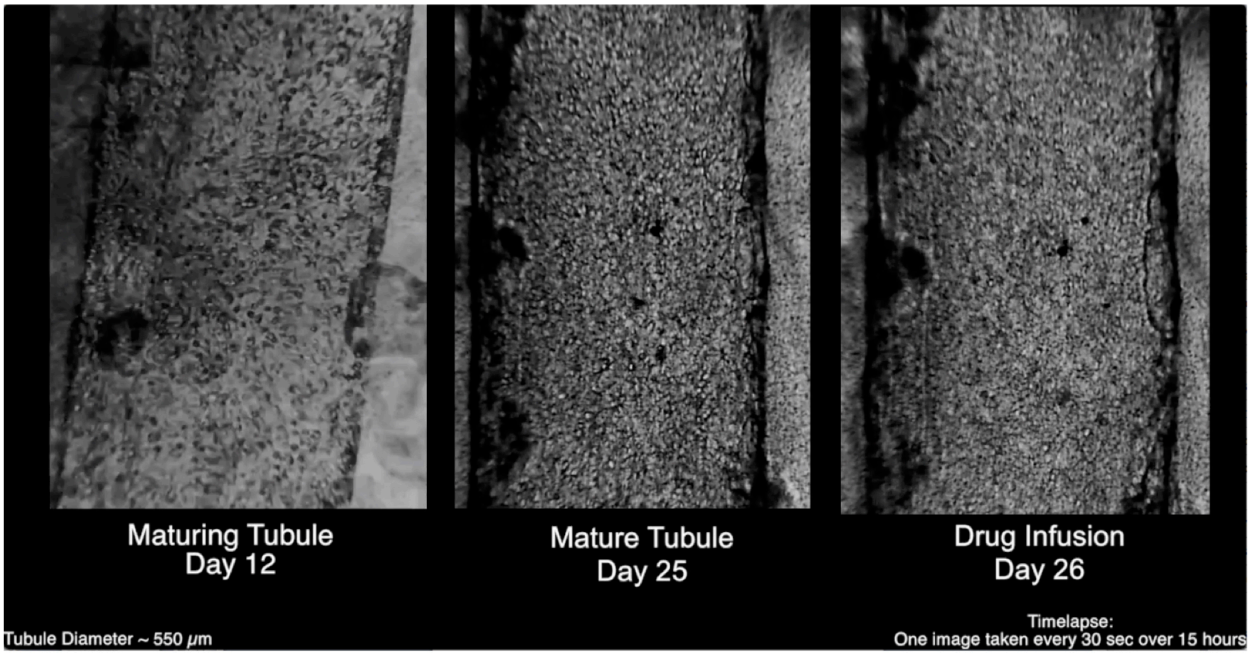

Movie S2. Movie of printed and perfused 3D proximal tubules during development and drug toxicity testing. Time lapse imaging of a 3D PT at Day 12 (left, while being fed 1% serum) and at Day 25 (middle, once serum was removed from media for 10 days), and during introduction of 50 μM Cyclosporine A (right). For each time lapse, images are taken every 30 sec for 15 h. The entire 15 h is played back over 45 sec.

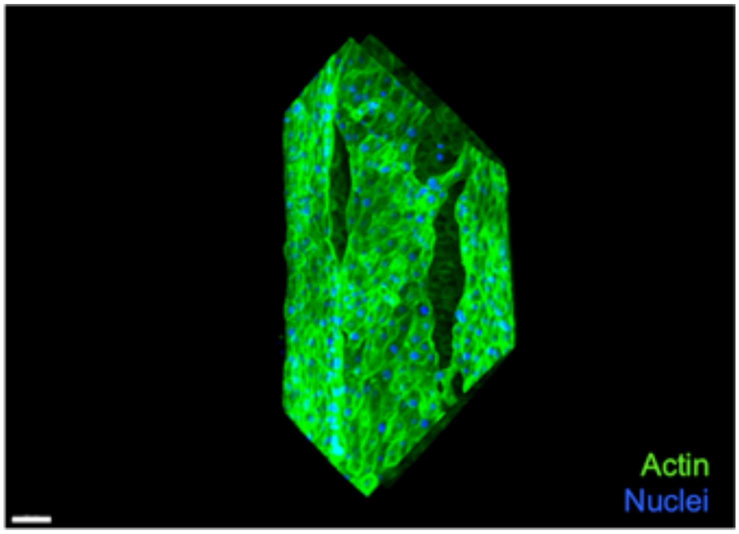

Movie S3. 3D rendering of a printed and perfused 3D proximal tubule after dosing with 100 µM Cyclosporine A for 24 h. The PT is stained with phalloidin and dapi to visualize actin filaments and cell nuclei, respectively.
